# The Impact of Socialisation on Children’s Prosocial Behaviour. A Study on Primary School Students

**DOI:** 10.3390/ijerph182212017

**Published:** 2021-11-16

**Authors:** Antonio Tintori, Giulia Ciancimino, Rossella Palomba, Cristiana Clementi, Loredana Cerbara

**Affiliations:** 1 Institute for Research on Population and Social Policies of the National Research Council of Italy (CNR-IRPPS), 00185 Rome, Italy; giulia.ciancimino@irpps.cnr.it (G.C.); r.palomba@irpps.cnr.it (R.P.); loredana.cerbara@irpps.cnr.it (L.C.); 2 Fondazione Movimento Bambino ONLUS, 00198 Rome, Italy; clementi.cristiana@gmail.com

**Keywords:** prosociality, socialisation, gender roles, stereotypes, cyberbullying, grooming, emotions, COVID-19

## Abstract

Background: Studying prosociality in children is a complex but relevant issue related to the qualitative development of human interactions. The main objective of the present study is to identify the psychosocial factors that most promote or inhibit the adoption of prosocial behaviours among children. Method: In Spring 2021, a survey was conducted amongst primary school children through a structured paper questionnaire. The data analysis has been carried out through bivariate and multivariate statistical techniques. Path analysis has been used. Results: The results highlight the role played by the parental education level, the perception of positive and negative emotions, the adherence to gender roles and the involvement in cyberbullying actions in predicting prosocial tendencies among children. On the other hand, adopting prosocial behaviours affects the screen-time as well as the devices’ interference in face-to-face interactions and the attitude towards school. Conclusions: The results are relevant and useful for the study of trends in prosocial behaviours among children. Family education level, individual status, peer interactions and social conditionings are variables that highly influence this multidimensional phenomenon. Further research is needed, including the definition of new measures and indicators concerning the context where children live and interact with others, with the aim of designing interventions aimed at facilitating relational well-being of children.

## 1. Introduction and Objectives

Prosociality refers to individual behaviours aimed at benefiting other people. The interest of scientific research on prosocial behaviours dates to the 1960s and 1970s of the last century, when the struggle for civil rights and humanitarian issues were at the centre of the socio-political scene [1]. Since then, studies on this topic proliferated with the aim to understand the mechanisms underlying prosocial actions and to clarify the differences between prosociality and other concepts closely related to it, such as altruism and empathy.

Today it is still difficult to find a common definition of prosociality because empirical research approached the concept in various ways, highlighting in turn some forms of prosocial tendencies rather than others. The lack of a single definition lies in its complex and multidimensional nature. Prosocial behaviours can take several forms, including informing, comforting, sharing, and helping [2]. On the other hand, the mechanisms of its development and functioning lie in many environmental and individual factors such as primary and secondary socialisation, embedding social norms and moral values, emotional and cognitive development but also biological characteristics [3,4]. For this reason, the disciplines that investigate prosociality are numerous and include psychology, philosophy, biology, psychiatry, and sociology, adopting different perspectives and methodologies. One of the broadest definitions of prosociality describes it as a set of actions that are intended to aid or benefit another person or groups of people without the actor’s expectation of external rewards [5]. Prosociality is also defined as a tendency involving actions characterised by the beneficial effects affecting others, highlighting the difference with altruistic behaviours. In this perspective, altruism belongs to the sphere of feelings and values oriented towards the good of others. However, being altruist does not necessarily mean acting prosocially because prosociality belongs to the sphere of social interaction’s practices. Therefore, prosocial behaviours are not always determined by altruistic motivations as they can give rise to intrinsic rewards for those who adopt them and depend more on the effectiveness of the actions [6].

Prosociality is also identified with “those behaviours that, without looking for external rewards, help other people or groups, according to the criteria of these. It also increases the probability of generating a positive reciprocity, solidarity and quality of interpersonal relationships or social consequences, safeguarding the identity, creativity and initiative of individuals or groups involved” [7]. In this case, the importance of the recipient of prosocial actions is emphasised as the atmosphere of reciprocity can engenders virtuous circles promoting individual and collective well-being. The reduction of depression and aggressive behaviours [8] as well as the promotion of academic success and the well-being of children and adolescents [9] are among the most important positive effects triggered by prosociality.

Most studies on prosocial behaviours investigated their development and correlates among young children and adolescents. However, some research has highlighted numerous benefits in terms of health, well-being and social inclusion that affect the entire life [10]. Prosocial tendencies in children reveal themselves from the first months of life [11] and vary with age according to individual experience and innermost characteristics [12,13]. According to numerous studies, prosocial behaviour has a positive relationship with age. It increases during adolescence compared to childhood, remaining stable during adulthood [14,15].

Cognitive development allows a better identification of needs and emotions but also fosters the detachment from an individual dimension approaching the perspective of others. In fact, the development of empathic skills allows us to understand needs and desires of others, which are a prerequisite for the adoption of a prosocial behaviour. As age increases, the capability of judgment and self-assessment and the adherence those values promoting prosocial conduct also increase [16]. According to other authors, it is not possible to trace a precise course of the prosocial behaviour’s development. This is mainly due to the complexity of its nature and the diversity of its forms, but also to the numerous methodologies used to investigate it and the strong influences from individual and environmental factors [17].

A study that analysed the relationship between cognition and behaviour in preschool and school children showed that as age increases along with cognitive abilities, the ability to differentiate among recipients of prosocial actions also increases. However, the study shows that with increasing age, discrimination and prejudices towards others also increase. Thus, the way these skills are shaped or channelled has relevant consequences for the evolution of society [18]. In addition, it appears that prosocial influence, i.e., the activation of more prosocial behaviours because of learning those behaviours from others, decreases as age increases. Children and adolescents are more susceptible than adults to social influences. Therefore, it is necessary to promote prosociality from childhood [19]. Finally, the time that children and adolescents spend in front of screens affects their emotional and behavioural development. According to several studies, as screen time increases, the likelihood of adopting antisocial and aggressive behaviours also increases, especially with a prolonged exposure to violent content [20]. Likewise, long-screen exposure is negatively associated with prosociality, especially when it comes to passive viewing (e.g., TV use) [21]. Moreover, spending more time in front of screens reduces parent-child interaction, resulting in less prosociality [22]. Other studies have highlighted the inadequacy of much research on screen time due to the use of poorly standardised outcome measures. In particular, to identify the effects of the exposure to violent video games on children and adolescents, it was shown that violent video games increase aggression and reduce prosocial behaviour [23].

With reference to sex differences in prosocial behaviours, the literature reports a greater prosocial tendency among females rather than males [15]. These results have been contradicted by other studies that did not find significant differences between the two sexes or that even found more prosocial behaviours among males rather than females [24]. This inconsistency in the results lies in the different methodologies used to investigate prosocial behaviours, and in the numerous forms of prosocial behaviour. Indeed, some studies have shown that females are more prosocial when referring to behaviours that involve caring for the other, while males are more likely to engage in public and heroic prosocial behaviours [25,26]. In this regard, there are two types of ethics orientations, the ethics of justice and rights and the ethics of care, which translate into different social behaviours: males would be more likely to behave prosocially against injustices and abuses, while women would mainly offer emotional support [27]. When analysing gender differences in relation to prosociality it is necessary to consider the type of prosocial behaviour under investigation together with the situational factors. This different propensity can be linked to the adherence to gender stereotypes learned during the process of primary socialisation [28,29,30]. During the secondary socialisation process, rigid social gender roles are often confirmed [31,32,33]. These social conditioning promote the feminine propensity to support and care for others, offering males heroic behavioural models to be embedded in case of dangerous situations [17,34]. In addition, gender is a relevant variable when referring to prosocial behaviours adopted by victims of cyberbullying [35]. According to numerous studies, victims of cyberbullying implement more antisocial strategies than non-victims facing a greater risk of becoming actors of cyberbullying [36,37]. Furthermore, gender differences related to cyberbullying reaction strategies have been highlighted, showing that these strategies are more prosocial in female victims than in male victims [35].

Regarding the relationship between prosocial behaviours and the socioeconomic background, in terms of parental education and income, there are many studies reporting dissimilar results [38]. The finding of some studies in the field of psychology have shown that having a low socioeconomic status predicts more prosocial behaviours such as generosity, trust, and availability, thanks to greater empathy as adaptive response to a more hostile social environment [39,40]. In contrast, recent studies have found positive relationships between high socioeconomic status and more prosocial behaviours among children and adolescents. People with a high socioeconomic background would have a better chance of satisfying their own emotional needs and receiving a less severe and punitive education than poorer children-factors that are often associated with more aggressive and antisocial behaviours [41,42,43]. Even in this case, the inconsistency of the scientific results about the influence of socioeconomic status on prosocial behaviours is related to the methodologies of investigation [44,45].

Finally, the relationship between perceived emotions and prosocial behaviours is multifaceted. Studies investigating how negative and positive emotions affect individual behaviours and choices highlighted that positive emotions promote prosociality and altruism [46]. The influence of negative emotions on behaviour appears more controversial. For example, sadness and anger have opposite effects on prosocial behaviours, while the former predicts them negatively, the latter promotes them [47].

Following Müssen and Eisenberg [5], we define prosociality as voluntary actions that are intended to benefit other people. Everybody knows that COVID-19 pandemic has made the virtual world ever more important, and children are spending an increasing time in front of screens from an early age. Although the study of childhood prosocial behaviour is not new, it should be noted the scarcity of statistical and quantitative studies on this age group. Therefore, it was decided to investigate prosociality among primary school children also considering the possible impact of the domestic isolation due to the COVID-19. The restrictive measures adopted to contain the pandemic spread have suddenly shifted most of the face-to-face interactions to a virtual space with serious repercussions on the well-being of the population and long-term effects which are still unknown. In particular, children and adolescents had to cope with the domestic isolation and the prolonged school closures which drastically reduced their activities outside the domestic sphere [48,49]. According to the results of many studies about the psychosocial impact of COVID-19 on this age group of the population, there was an increase of anxiety, depression, irritability, loneliness and fear [50,51,52,53,54,55,56,57].

In Spring 2021, we carried out a survey on primary school children with the aim to study prosociality and the development of prosocial skills in early ages. Due to the very young age of the target population, we measured prosociality in terms of the capacity to understand the emotional status of others, to share personal emotions, and to adopt helping behaviours.

The aim of the present study is to identify the psychosocial factors which influence or are influenced by prosociality. The study focused on both individual variables and variables related to the social context of respondents, with the aim of understanding better the relations between socialisation process and development of prosocial skills. We hypothesised that some types of socialisation factors, as for example the family background, the interaction among peers, the presence of rigid cognitive schemes [58], risky or deviant on-line behaviour as well as individual emotions may influence the level of prosociality of children. We consider sex an important factor in determining different levels of prosociality. Finally, we hypothesised that the level of prosociality in turn influences face-to-face interactions and attitudes towards the school.

## 2. Methodology

In Spring 2021 we conducted a survey on primary school children. The survey involved children from two large Rome areas: the Sixth and Eighth Districts. The city of Rome is divided into fifteen districts which are called *municipi*. Each of these districts has different territorial extensions and population densities. The two selected areas represent respectively 9% and 5% of the resident population of Rome. The 6th and 8th districts are characterised by similar population density (between 2000 and 3000 inhabitants per km^2^) and heterogenous demographic and economic profiles. Together they have almost 400,000 inhabitants, that is the equivalent to the population of a large city. We selected areas with dissimilar socio-economic profiles to limit bias in estimates due to the excess of homogeneity in the population. A sampling plan representative of the two districts was carried out, taking into consideration all primary schools present in the areas. In total, eight schools were selected, four for each district. Permit to collect data and enter the school premises was requested to school’s managers and representatives by telephone firstly, then by e-mail, outlining the relevance of scientific topics and objectives.

The current COVID-19 pandemic and the measures to prevent the spread of the virus were an obstacle in granting research team physical access to school buildings. While speaking with schools’ managers, researchers underlined the importance of their physical presence during the administration of the questionnaires to ensure a better control and quality of the data (complete autonomy of interviewees in choosing the answers, better understanding of the questions, and minimisation of mutual and adult influence). The permit to access school classrooms to carry out the survey was obtained, because school administrations recognised the importance and urgency of the study. In fact, the COVID-19 pandemic and the consequent distancing measures reduced physical interactions and may have produced a dangerous shift from social relations into virtual world among the youngsters.

### 2.1. Participants

The survey was carried out among students attending the last three years of primary school; the classrooms to be involved in the survey were selected by school administrators. The administrators choose one class from each school grade to ensure the complete age groups coverage. The activity took place between 14 April and 13 May 2021. In total, 412 face-to-face interviews were collected. The sample was composed of 46.3% females and 53.7% of males. A total of 35.4% were attending the 3rd year of primary school, 31.7% the 4th year and 32.9% the 5th year. The data were collected through a structured questionnaire, pre-tested on 40 children aged between 8 and 11 years.

### 2.2. Data Collection Tools

The questionnaire design was carried out on the basis of a research project agreed with the Presidency of the Council of Ministers of Italy, who co-financed the survey. The research design included the study of specific themes and social issues.

It was a paper questionnaire consisting of 42 questions. Due to the young age of respondents, questions were laid out with considerable caution; special attention was paid to the font type and size. The questionnaire covered the following main topics: quantity and quality of social interaction among peers; time spent in front of screens, cyberbullying, sexting and online grooming, psychophysical well-being, gender stereotypes and roles, and prosociality. The questionnaire construction has started from the analysis of the existing literature about social interaction, discomfort and deviance concerning this segment of the population. In addition, it was built through the investigation techniques historically used by the research group to study human behaviours and attitudes. In the light of the research design, the following main dimensions of analysis were selected:

− Quantity and quality of social interaction among peers;
− Screen time;
− Cyberbullying, sexting, online grooming;
− Well-being;
− Social attitudes and behaviours.

The operationalisation process necessary to turn abstract concepts into variables and questions able to measure the phenomena mentioned above was very complex. Indeed, this process had to consider the possible effects of the spread of Cov-19 on attitudes and behaviours of young people, especially those of the domestic confinement and the reduction of physical interactions.

The main variables adopted were:

− Vertical and horizontal social interactions (leisure, sports, relational trust);
− Online events and behaviours;
− Electronic devices used and purposes and time of use;
− Favourite video games;
− Favourite social media;
− Individual status in relation to the use of electronic devices, video games and social media;
− Prosocial tendencies and behaviours;
− Gender stereotypes.

### 2.3. Procedures

The administration phase has been led by two researchers, one of whom was the project leader, with a long experience in assisting the youth population during the compilation of a questionnaire, instead of relying on external interviewers. Their presence in each class was crucial to ensure the widest understanding of the questions by all the students who answered individually. Furthermore, in this way it was possible to limit teachers’ interferences, minimising the risk of bias deriving from their presence. On average respondents took 35 min to answer all the questions.

From the operational point of view, a package containing informed consent forms was physically delivered to each school at least 20 days before the day of the survey. These forms were necessary to obtain parental consent to interview children; the parental consent form was accompanied by a family socio-demographic form, containing questions about the number of cohabiting family members, the number of cohabiting and non-cohabiting brothers and sisters, citizenship, marital status, educational qualification, and employment status. The information about families were then associated with the students’ questionnaire by the schoolteachers through a numerical code to guarantee the anonymity of the participants. This procedure guarantees the privacy of the interviewees, and it was carried out thanks to the collaboration of the schools; it allowed the research group to associate the socio-demographic information on families to the data collected among children. In fact, it is well known that the family context plays a key-role in determining specific children’s attitudes and behaviours.

### 2.4. Data Analysis

The analysis of the research results was carried out using the SPSS software (version 26 server) (IBM, Chicago, IL, USA), through bivariate statistical processing and subsequently using multivariate analysis techniques, in particular LISREL causal models [59] which were applied to variables and quantitative synthetic indicators. For the purposes of the study, a specific prosociality indicator was built to measure the individual propensity to benefit other people. This indicator was based on three variables concerning the adoption of prosocial behaviours in different situations. These variables have been operationalised into three multiple choice questions that investigated opinions and behaviours adopted by respondents. More specifically, participants were asked to express their opinion about: the utility of understanding the other’s feeling, their favourite way to compliment a friend, and how they try to understand what a friend feels. For each of these questions there were four possible answers linked to the presence of prosocial tendencies, a neutral behaviour, a self-centred tendency, and indifference [Appendix A]. Analysing the collected answers, three partial indicators were calculated, one for each of the questions indicated, with 4 levels of prosociality: low, medium–low, medium–high, and high. Finally, the three partial indicators were synthesised in an average value to be used as continuous variable in the regression models [60,61]. The three partial indicators were also reduced into a single indicator of prosociality with 3 classes—low, medium, and high—to facilitate bivariate analysis.

Additional indicators were also built for both the descriptive bivariate analysis, and multivariate analysis. For example, the parental educational level indicator was built on the parents’ educational qualification collected in the family socio-demographic form. Considering simultaneously the answers of both parents, 6 pre-coded modalities of response were synthesised to build an indicator consisting of 4 levels of parental education: low, medium–low, medium–high, and high. In the same way, the parental employment status indicator was built, through a question about the parental employment situations. The outcome of the recoding process was a 4-level employment status indicator: low, medium–low, medium–high, and high. Two indicators about the perception of positive and negative emotions were built on the basis of the frequency of the perception of specific emotions. Respondents were asked to indicate the perception’s frequency of the following emotions on 4-point scales from “never” to “always”: anger, sadness, fear, loneliness, anxiety, happiness, and calm. These two indicators are the result of the sum of the answers collected, dichotomised during the recoding process between never/almost never and always/almost always. Moreover, to investigate the online deviant behaviours a list of actions was submitted to the respondents, such as to quarrel, to insult, to threat, to exclude someone from a group, to share photos or videos of someone without consent. Those who had carried out at least one of these actions were defined cyberbullying actors, while those who have suffered at least one of these actions were considered cyberbullying victims. Finally, those who have suffered specific online actions carried out by adults were considered grooming victims. The indicators about the adherence to male and female roles, was built on the basis of a variable designed to measure the internalisation of stereotyped behavioural patterns. In this case, a list of actions and roles was submitted to the participants asking who they think could carried out better: men, women or if sex was irrelevant. Analysing the collected answers, the adherence of respondents to stereotyped male and female models was calculated and 4 levels of adherence to gender roles were identified: absent, low, medium, and high.

Finally, the screen-time indicator was built from four variables investigating the time spent playing video games and using social media and applications. The frequency was collected in days per week and in hours per day. Firstly, two indicators were built, one about the time spent on video games and one about the time spent on social media and applications. The outcome of the recoding process was the screen-time indicator, which synthetises these two indicators and identifies four levels of screen-time: absent, low, medium, high.

In the analysis of data, we used the path analysis technique which is a method for studying the direct effects or indirect effects of variables with the aim of designing a model of reciprocal interactions. Path analysis is a straightforward extension of multiple regressions [60,61]. It provides estimates of magnitude and significance of hypothesised causal connections between sets of variables. In our study, we used path analysis to highlight the existence of relationships between the indicator of prosociality and other collected variables or indicators (we used the recursive scheme proposed by Lomax [62]). In fact, the task of path analysis is to ascertain whether there is a meaningful pattern among data and statistically design a model of the possible patterns of direct and indirect relations between data.

**Figure 1:**
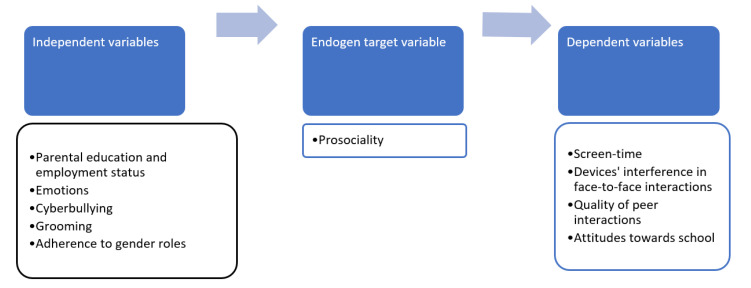
Theoretical path model concerning prosociality indicator.

The prosociality indicator was considered as endogenous variable because we hypothesised that it can be dependent on some variables and independent from some other variables. Figure 1 shows the theoretical approach. In the centre of Figure 1, there is the indicator of prosociality tested both as dependent variable for the identification of its possible determinants, and as independent factor, for the identification of its possible impact on other variables related to the respondent’s behaviours. The results will be showed through the standardised β-values for evaluating the correlation between the variables involved in the model. Only significant relationships will be illustrated.

## 3. Results

### 3.1. Results of the Bivariate Statistical Analysis

**Figure 2:**
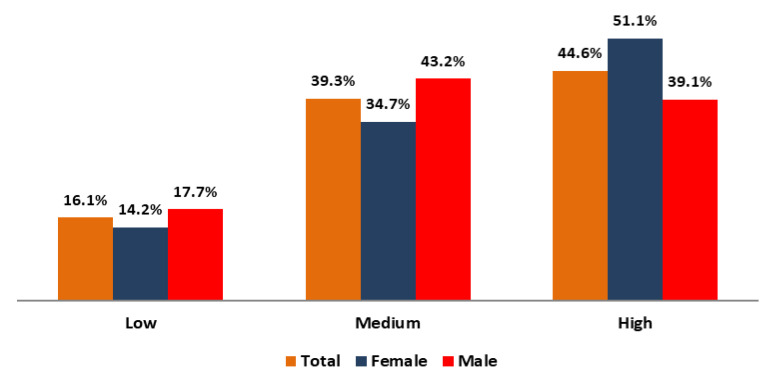
Prosociality levels (% by sex).

The level of respondents’ prosociality measured through the indicator described above was 44.6% high, 39.3% medium and 16.1% low. In relation to sex, a greater prosocial tendency was found among girls (51.1% of females showed a high prosocial level against 39.1% of males) (Figure 2). Significant differences were also found in relation to the socio-economic environment. The lowest levels of prosociality were found among respondents from schools located in the Sixth District of Rome, a critical context from a socio-cultural and economic point of view (37.4% as compared to 51.2% of the Eighth District, that is more advanced in terms of general level of education and employment status). Furthermore, the level of parental education was a relevant variable in relation to the adoption of prosocial behaviours among respondents. In fact, students with parents having a high educational level showed greater prosocial tendency (53.5% against 35.8% of those from families with a low level of education).

Greater prosociality was also found among respondents who spend their free time with friends, meeting them in person (49.1%), and with brothers and sisters (49.1%). On the other hand, only children showed less prosocial tendencies than children with brothers and sisters (a high level of prosociality was found in 36.4% among only children and 46.2% among respondents with siblings). Prosociality decreases for students who spend their free time alone (40%) and even more for those talking with friends online (39.2%). To confirm this trend, a higher level of prosociality was found among those who prefer to talk with friends in person (45.5%) rather than exclusively in chat (35%). This is a very interesting and alarming result, considering the growing spread of the virtual social interaction, also due to the spread of COVID-19. In fact, the most hyper-connected students who spend a lot of time on social media and videogames are those with a lower level of prosociality (among them only 34.8% showed a high level of prosociality against 49% of respondents with a low level of screen-time). In particular, the results show that those who use violent video games have a lower level of prosociality than those who use other types of video games (30.5% against 48.4%).

Prosociality increases for students who described their own friends as affectionate (52.4%) and peaceful (45.5%), while it decreases when friends were described as quarrelsome (40.5%). In relation to the sports practice the question investigated the access to out-of-school sport before the COVID-19 pandemic. Results showed that respondents who practiced sport before the pandemic had a high level of prosociality (47.4% of cases as compared to 34.2% of those who did not). Moreover, in terms of social participation, a high level of prosociality was found especially among children with both Italian parents (46.2%), who are maybe more socially integrated compared to those who come from families with one foreign parent (40.9%) and to those with both foreign parents (27.3%), while a lower prosocial tendency was found among those who wish not to go to school. In addition, trust in parents and relatives, more than in friends and teachers was related to greater prosociality. This could confirm the importance of the family context in determining positive social behaviours. Among the other variables linked to the adoption of prosocial behaviour, students who have been involved in cyberbullying actions have lower level of prosociality than the other students. Among cyberbullying actors 39.3% showed a high level of prosociality, against 48.1% of the other students, while among victims 32.9% showed a high level of prosociality against 49.8% of non-victims. Finally, the adherence to male and female gender roles inhibits prosocial behaviours: among respondents who strongly believe in the existence of male gender roles 36.7% showed a high level of prosociality against 55.2% among students who do not believe in these social conditioning; among respondents who adhere to female gender roles 35.9% showed a high level of prosociality against 55.7% among students who do not believe in these social conditioning.

### 3.2. Results of the Path Analysis Model

In carrying out path analysis the collective was divided by sex. The indicators involved in the model were the following: the indicator about the parents’ educational level, the two indicators that measure adherence to gender roles, which refer respectively to male and female roles, the indicator about the frequency of the perception of negative and positive emotions, and finally the indicator regarding the actions of cyberbullying and grooming suffered or acted by the respondents. We also introduced two additional statistically significant variables: the devices’ interference that is using media such as phone or video chatting, listening to music, and gaming, while interacting with others during face-to-face interactions and the level of enjoying attending school.

**Figure 3:**
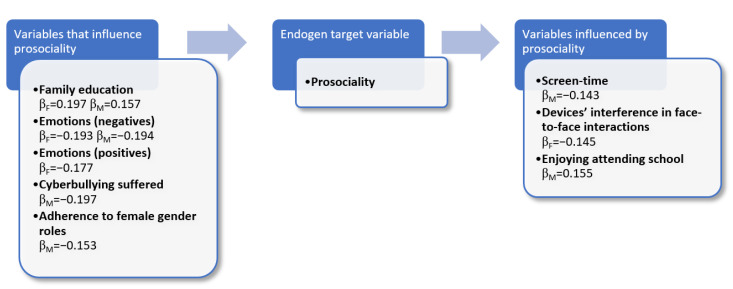
Results of the path models related to prosociality.

In Figure 3, the results of path analysis are shown. Indicators and variables with the highest β are those that influence, or are most influenced, by prosociality. To be noted that standardized coefficient β is β_F_ for females and β_M_ for males, because we divided our sample by sex.

The model shows differences between girls and boys. Factors influencing prosociality are listed on the left of Figure 3; on the right side the factors influenced by prosociality are shown. The results of the path model indicate the existence of direct correlation between parental education and the level of prosociality. As the level of education of parents increases, the level of prosociality of both males and females increases. The level of prosociality decreases with increasing perception of negative emotions of respondents. For female respondents, the level of prosociality increases with the increase in perception of positive emotions (β_F_ = −0.177), such as calm and happiness. For male respondents having suffered acts of cyberbullying or grooming online the level of prosociality decreases. Finally, in the case of male respondents, the adherence to female gender roles is detrimental to prosocial behaviours (β_M_ = −0.153).

The model has been completed by using the prosociality indicator as independent variable to better understand its effects on the attitudes and behaviours of the respondents (Figure 3). According to the results of the model, a high prosocial tendency reduces the screen time for male respondents, while for females it reduces the risk of devices’ interference in face-to-face interactions. Furthermore, the high prosociality level of males affects their pleasure in attending school.

## 4. Discussion

A clear and deep understanding of the mechanisms of prosociality can be a complex undertaking as there are many individual and social factors related to it. The study of prosociality implies an in-depth analysis of the social context of the observed subjects, such as the economic and cultural status of the family of origin, the composition of the peer group and the ways of social interaction. However, from this perspective, the present study highlights significant trends showing predictive factors and effects of the levels of prosociality among children.

Sex is an important factor in determining prosociality, because of the stereotyped socialisation which leads girls to pay more attention than boys to prosocial behaviours, especially in terms of care of the others. Another relevant variable is the parents’ educational level, which in turn affects the time children spend in front of screens. Indeed, interaction and social inclusion play an important role in predicting greater prosocial tendency. In this regard, results of the bivariate statistical analysis show that respondents who are more solitary and prefer to talk with friends online adopt less prosocial behaviours.

Due to the spread of COVID-19 and related containment measures, this tendency is a serious risk of isolation that should make us think very carefully about what may happen in the near future. Our conclusions are in line with previous studies [20,21] concerning the likelihood of antisocial and even aggressive behaviour with increasing time spent by children on front of screens. Our data show that it is possible that the exposure to specific on-screen stimuli, such as violent video games, leads to a lower level of prosociality. To be noted that we also found that children with low prosociality level tend to spend more time in front of a screen and not vice versa. We hypothesised that this is the effect of different primary socialisation due to the level of parental education, which represents a very influential variable on prosociality of children. We believe that the educational level, and not the economic condition, affects specific phenomena such as prosociality. The lack of standardised research methodologies in this field may lead to existing interpretative differences in prosociality.

Our study confirms that positive emotions promote prosociality in girls. About the perception of negative emotions, our data disprove that sadness and anger have both opposite effects on prosocial behaviours [47]. We found that anger reduces prosociality, though the relation is very weak. Deviance is usually associated with antisocial behaviours and our results show that actors and victims of cyberbullying have a lower prosocial tendency. Females remain more prosocial even when victims of cyberbullying. To be remembered that we investigated on a group of very young children and there is the need to designed common effective indicators to measure prosociality within specific population groups.

## 5. Conclusions

The study presented is based on a survey conducted in Rome, Italy, during the spring of 2021. The survey was carried out on a sample of children attending primary school through a paper questionnaire. The results are useful for studying the development of prosocial behaviours in young ages. The survey is a positive experiment both in terms of feasibility and participation in surveys of schools and students but also in terms of duration, costs and adequacy of the survey tools. The showed importance of family, individual status, cyberbullying phenomena and social conditioning on prosociality, as well as the influence of prosociality levels on the screen time, face-to-face interactions and attitudes towards school, suggest the need for further studies on a larger geographical scale with a statistical sample representative of the Italian primary school children. A larger sample will allow carrying out even more detailed statistical analysis and a more complex profiling of the participants. In the field of social sciences, a progressive standardisation of research methodologies and a wider use of both sociological and psychological variables is desirable to analyse more in depth the correlates of the behavioural problems of childhood.

It is very likely that the influence of variables such as age, sex and socioeconomic background on prosocial behaviour, screen time, is controversial due to the different methodologies adopted in studying this phenomenon among children and adolescents [17,23,44,45].

Detecting and understanding relational and behavioural dynamics is certainly necessary to define effective social policies and targeted interventions. This is crucial to stem both endemic social pathologies and those induced by the spread of COVID-19. The pandemic has strongly conditioned, and will continue to condition, interactions amongst young people. Therefore, reaching an advanced and increasingly shared knowledge of these phenomena is necessary to promote the well-being of children, to provide them critical and ethical skills in the use of modern tools of communication and interaction, and create greater possibilities for social inclusion. It implies the support of formal and informal relational networks, for example through the promotion of sports practice as an element enabling socialisation, but also the quality of social relationships. However, this study represents the first step in a larger project commissioned by the Department for Family Policies under the Presidency of the Council of Ministers of Italy. In the light of the obtained results, the costumer will define educational policies and interventions addressed to parents, teachers and students with the aim of promoting the well-being of the youth population. In this way, it could be possible to counteract the hyper connection, the antisocial behaviour as well as the reproduction and increase in social distances in favour of greater prosociality.

## Data Availability

Data of this study are not available because of the co-property with the Italian Presidency of the Council of Ministers.

## Appendix A. Additional Materials

**Table A1:** Questions and pre-coded responses on prosocial tendency among children.

**1.** **In your opinion, why is it useful to understand what a person feels, even if he/she does not tell you?**
I can help
I can avoid bothering him/her
I can steer clear from him/her to avoid trouble
It is useless, it is not my business
**2.** **In your opinion, why is it useful to understand what a person feels, even if he/she does not tell you?**
Make a kind action
Tell him/her in person
Text him/her
Just think about it, because I am ashamed to say it openly
**3.** **When you are with a friend, how do you know what he/she does feel, if he/she does not tell you directly?**
From the tone of his/her voice
From his/her posture
From his/her facial expression
From the gestures he/she makes
